# Rib Healing and Heterotopic Ossification After Surgical Stabilization of Rib Fractures

**DOI:** 10.3390/jcm14155581

**Published:** 2025-08-07

**Authors:** Alexander Hoey, Daniel Akyeampong, Arjun Patel, Ronald Gross, Evert A. Eriksson

**Affiliations:** 1 Department of Surgery, Medical University of South Carolina, Charleston, SC 29425, USA; hoeya@musc.edu (A.H.); akyeampo@musc.edu (D.A.);; 2 Department of Surgery, Trinity Health of New England, Hartford, CT 06105, USA; rigross1202@gmail.com

**Keywords:** rib fracture, non-union rib fracture, hypertrophic ossification, surgical stabilization of rib fractures, anatomy

## Abstract

**Background**: Little is known about the rate of rib fracture healing after Surgical Stabilization of Rib Fractures (SSRFs). We sought to evaluate the radiographic evidence of rib healing and hypertrophic ossification (HO) in patients after SSRFs. **Methods:** A single-center retrospective cohort study was conducted on all patients who had undergone SSRFs from 1 January 2010 to 31 March 2023 and had a computed tomography (CT) of the chest performed greater than 6 months after SSRFs. The rib fracture locations were mapped on the initial trauma CT scan and evaluated on the follow-up CT scan for healing and HO formation. **Results:** A total of 254 SSRF cases were evaluated, 21 patients met the inclusion criteria; out of 208 fractures, 109 underwent SSRFs. The median time to follow-up CT scan was 17(7–88) months. Overall, 95% of the fractures healed completely. Seventy percent of the non-union fractures were in posterior or paraspinal locations on ribs 8–10. HO was noted in nine patients and seen as early as 8 months post-operatively. A significant association was identified between the fixation method used to perform SSRFs (89% vs. 11%, *p* = 0.024) and operative day (6(0–9) vs. 2(2–5), *p* = 0.023). **Conclusions:** Non-union of rib fractures is uncommon after SSRFs. Many of these fractures involve the posterior or paraspinal lower rib cage. HO between fractures is common after SSRFs.

## 1. Introduction

In recent years, surgical stabilization for rib fractures (SSRFs) in cases of thoracic wall trauma has witnessed a notable surge [1]. This increasing trend is largely attributed to advancements in surgical techniques and an expanding body of research that supports the benefits of SSRFs in specific patient populations. The recent literature has demonstrated that, when compared to non-operative or traditional conservative management, SSRFs may lead to several clinically significant improvements. These include a reduction in narcotic pain medication use, shortened intensive care unit (ICU) stays, fewer days spent on mechanical ventilation, and even decreased mortality rates in some cohorts [2,3,4]. These findings suggest that the SSRFs not only addresses structural integrity of the chest wall but also contributes meaningfully to improved overall patient outcomes.

Despite these promising outcomes, there remains a considerable gap in our understanding of the biological and radiological aspects of rib fracture healing following surgical stabilization. As SSRFs becomes more widely accepted, it is increasingly important to study and clarify the underlying mechanisms of post-fixation bone healing, which is a domain that is still not fully elucidated. Greater insight into this healing process could aid in optimizing surgical strategies, reducing complications, and improving long-term functional outcomes for patients.

Bone healing, in general, is a highly coordinated and complex physiological process. It encompasses multiple overlapping stages, including an inflammatory phase, a reparative phase characterized by new bone formation, and a remodeling phase that restores the bone to its original shape and strength. These stages are influenced by several critical factors such as biomechanical stability at the fracture site, adequate blood supply, and the local cellular environment [5]. When bones are properly aligned and stabilized—as is the intent with surgical fixation the healing process is more likely to proceed in a predictable and efficient manner. The application of internal fixation devices such as plates and screws provides mechanical support that limits motion at the fracture site, thereby promoting favorable biological conditions for bone remodeling [6].

However, when the healing environment is suboptimal—whether due to inadequate stabilization, poor vascularization, or patient-specific factors such as smoking or metabolic disease—complications such as non-union can arise [6]. Non-union refers to the failure of a fracture to heal within the expected timeframe, and it can lead to prolonged pain, decreased respiratory function, and diminished quality of life. In the context of rib fractures, this complication is particularly significant as it can interfere with effective breathing and increase the risk of pulmonary complications.

In addition to non-union, another possible complication following trauma or surgical intervention is heterotopic ossification (HO). HO is the aberrant formation of bone tissue in locations where bone is not typically found, such as within muscles or connective tissues of the chest wall [6]. This abnormal bone growth often results from an exaggerated healing response and may be precipitated by trauma, surgery, or prolonged inflammation. In patients with rib fractures, especially those treated with SSRFs, HO can result in localized pain, stiffness, and in some cases, functional impairment of the chest wall.

Despite the clinical relevance of both non-union and HO in rib fracture management, data on their incidence, particularly in surgically stabilized cases, remain limited. Very few studies have rigorously quantified the frequency with which these complications occur following SSRFs. Moreover, it remains unclear whether surgical stabilization itself increases or decreases the likelihood of these outcomes. Understanding the role of fixation in either promoting or preventing non-union and HO is crucial for refining patient selection criteria, surgical techniques, and post-operative management protocols.

To address this knowledge gap, our study aimed to analyze the prevalence and radiographic characteristics of rib fracture healing and heterotopic ossification in patients who underwent SSRFs at our institution. By examining available radiological evidence, we sought to document and describe the patterns of bone union and the presence of HO in this patient cohort. Our findings may contribute to the broader effort of defining the risks and benefits associated with surgical stabilization, and may also support the development of strategies to mitigate potential complications.

This study was originally presented as a meeting abstract at the 2024 Chest Wall Injury Society Annual Scientific Meeting, held on 12 April 2024. This work represents an initial step toward a more detailed understanding of post-operative rib fracture outcomes and highlights the necessity for ongoing research in this evolving field of thoracic trauma care.

## 2. Materials and Methods

A single-center retrospective cohort study was conducted on all patients who had undergone SSRFs from 1 January 2010 to 31 March 2023. Institutional Review Board approval was obtained for this project at the Medical University of South Carolina (Pro00144366). All patients were required to have undergone a chest computed tomography (CT) scan prior to their SSRF as part of their trauma work-up, as well as a post-operative follow-up scan at least 6 months after their index case. Any CT scan performed on the patient greater than 6 months after injury was evaluated for signs of healing or HO. The CT scans were performed as part of regular care and were not part of routine follow-up of patients after SSRFs. The CT scans were obtained based on the clinical decision-making of the treating physician at the time. None were performed for symptoms associated with the initial injury or out of concern for hardware problems or failure.

**Figure 1:**
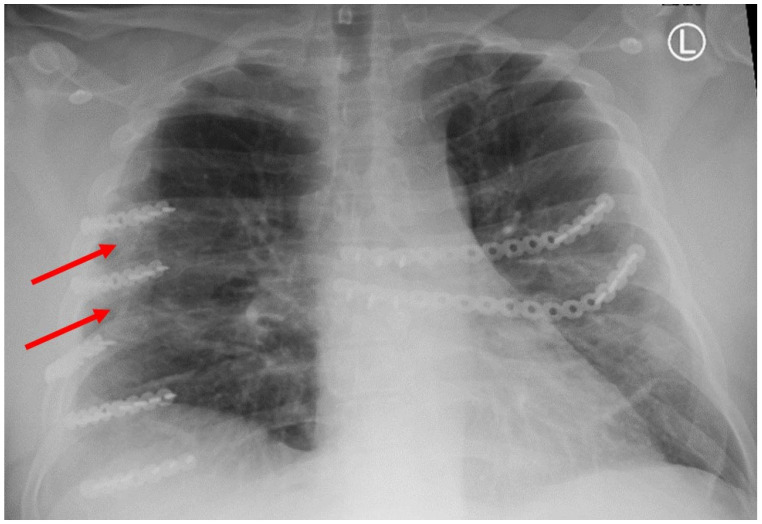
Chest X-ray showing HO at the tips of the red arrows.

**Figure 2:**
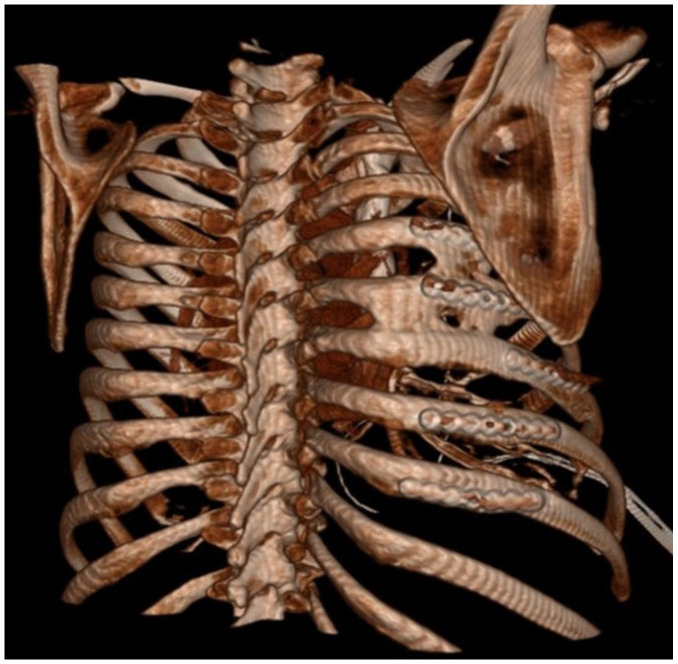
Three-dimentional reconstruction of CT scan with HO between ribs.

Patient-based variables were obtained from the medical records, including the following: age, race, gender, mechanism of injury, injury severity score (ISS), regional abbreviated injury severity score (AIS), medication administration, hospital length of stay, ICU length of stay, days of ventilator support, and discharge location. The existence of polytrauma was also evaluated, which was defined as any injury in a body region besides the chest with an AIS of 2 or greater. Rib fracture locations as well as displacement were characterized on initial CT scans based on their anatomical sector. These sectors consisted of the following: costal cartilage (CC); anterior (A) [rib costal cartilage junction to anterior axillary line]; lateral (L) [anterior axillary line–posterior axillary line]; posterior (P) [posterior axillary line to the lateral aspect of the paraspinus musculature]; and paraspinal (PS) [rib head–lateral aspect of the paraspinus muscles]. These anatomic divisions were used to further describe the location of SSRF titanium hardware on subsequent scans. All ribs were fixated using titanium plates with bicortical screw placement with a minimum of three screws anterior and posterior to the location of the fractures following the manufacturer’s guidelines. Both self-tapping (ST) and self-drilling (SD) screws were utilized as SD screws were not available until November 2019. ST screws require pre-drilling a bicortical osteotomy prior to placing the ST screw, which uses this pilot hole to guide its insertion into the rib. SD screws do not require this pilot hole to be drilled through the rib for fixation to occur. The number of plates used for fixation, as well as type of screws used for fixation, were recorded. Additionally, every patient undergoing SSRF underwent pleural space irrigation with 1 L of fluid and had a chest tube placed. Subcutaneous or submuscular drains were not used in any patients. All SSRF procedures were performed at a single center and by a single surgeon. Evidence of fracture healing and HO was also assessed using follow-up imaging evaluating the entire chest wall. The locations of the fractures as well as HO were characterized in the same manner as described above based on anatomic location. Non-union fractures were defined as a lack of cortical bone connection noted on follow-up CT scan axial cross-sectional imaging. HO was defined as calcium deposition in non-anatomic locations for bone (Figure 1 and Figure 2).

Data were analyzed utilizing the Statistical Package for the Social Sciences (SPSS), version 27 (SPSS, Chicago, IL, USA). The Shapiro–Wilk test was used to determine normality for continuous variables. Typically, distributed data are expressed as mean ± standard deviation, while non-normally distributed data are shown as the median with a range from minimum to maximum. A *p*-value of 0.05 or less was regarded as statistically significant. All statistical tests were conducted as two-sided analyses. For continuous variables, normally distributed data were evaluated with Student’s t-test, whereas non-normally distributed data were assessed using the Mann–Whitney U-test. For categorical variables, frequencies and proportions were compared using either the Chi-squared test or Fisher’s Exact test, depending on the context.

## 3. Results

A total of 254 patients were evaluated, 21 of whom met the inclusion criteria requiring a follow-up CT scan greater than 6 months after their index operation. None of the CT scans were obtained for concern of hardware failure, displacement, or bony chest wall symptoms. Average age at the time of SSRF was 62 ± 14 years, and 76% of the group were male. ST screws were used in 57% of patients (12/21). The median time to surgery was 3 (0–9) days, and the median time to follow-up CT scan was 17 (7–88) months. A total of 208 fractures (median/patient 8 (3–21)), with 107 plates and 2 intra-medullary splints (median/patient 5 (3–11)), were evaluated. The locations of fractures are shown in a heatmap in Table 1.

Overall, 95% of the fractures healed completely when evaluated on follow-up imaging. Ninety-five percent (102/109) of the fractures that were repaired with titanium support healed. Five percent (5/99) of fractures that did not undergo SSRFs had evidence of non-union. Seventy percent of the non-union fractures were posterior or paraspinal fractures on ribs 8–10. All of the non-union rib fractures were inferior to the area of the chest wall that underwent SSRFs, with the exception of one non-union fracture on rib 2 in one patient. All of the anterior and costal cartilage fractures healed normally. Three lateral fractures did not heal normally. The location of the non-union rib fractures is depicted in Table 2.

**Figure 3:**
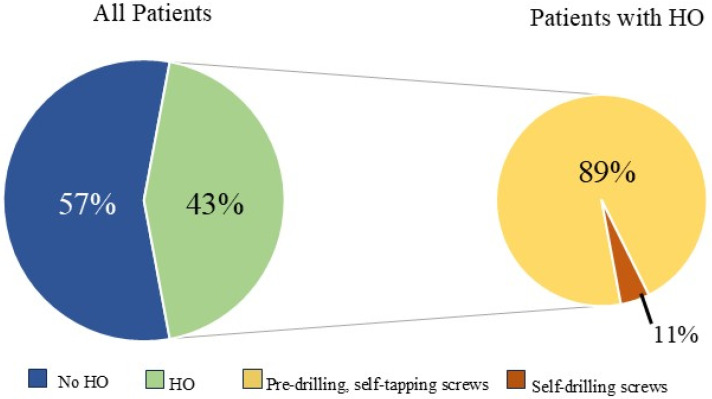
Relationship between heterotopic ossification (HO) development and screw fixation method.

HO was noted in nine patients (43%) and seen as early as 8 months post-operatively. Overall, 15% (32/208) of rib fractures showed evidence of HO at follow-up. In patients that developed HO, 36% (32/88) of rib fractures had evidence of HO formation. The HO was evident on posterior–anterior chest X-ray in 25% (2/8) of patients. No significant difference was noted in patients with HO when accounting for gender (43.8% vs. 56.3%, *p* = 0.647), ISS (24 ± 12 vs. 18 ± 9, *p* = 0.265), flail chest injury (36.4% vs. 63.6%, *p* = 0.425), number of fractures (8 (4–16) vs. 9 (3–21) *p* = 0.917), polytrauma (44% vs. 50%, *p* = 0.801),time to follow-up CT scan (18.0 (7.7–88.5) vs. 14.2 (7.1–56.7) *p* = 0.247), or number of SSRF plates (4 (3–11) vs. 5 (3–11) *p* = 0.862). The number of patients with traumatic brain injury was similar, with three patients in both groups. A significant difference in these patients was identified when comparing approach to screw fixation pre-drilling with self-tapping (ST) screws versus self-drilling (SD) screws (89% vs. 11%, *p* = 0.024), year of operation (2107 ± 1.9 vs. 2020 ± 1.7, *p* = 0.004), and operative day (6 (0–9) vs. 2 (2–5), *p* = 0.023). All patients were treated with non-steroidal anti-inflammatory medications after injury (ibuprofen). The difference in patients with HO and the difference in types of screws used are illustrated in Figure 3.

## 4. Discussion

The aim of this investigation was to determine the rate of fracture healing following SSRFs as well as to characterize the occurrence of HO after chest trauma. As SSRFs has become more commonplace, alternative rib plating systems have been developed with plates that vary in thickness, flexibility, absorbability, and fixation technique. There has been a growing interest in the performance and outcomes of different rib plating systems. Recent studies comparing efficacy of non-absorbable and absorbable plates note a significant difference in outcomes, highlighting a need to expand this investigation to a multi-center study comparing rib healing rates after SSRFs using various plating systems [7]. This underscores the importance of conducting a multi-center study that systematically compares rib healing across diverse plating systems, which would help establish evidence-based guidelines tailored to both patient-specific and injury-specific factors.

Our data also suggest fractures in the posterior and paraspinal rib sectors that are low in the rib cage are at highest risk of non-union. Interestingly, these locations correlate with the recent literature on rib fracture stability that suggests the posterior and paraspinal sectors are at the highest likelihood of progressive offset following injury [5]. The same group proposes that the unique biomechanical forces on these ribs and the basic rib structures may play a role in their displacement [5].

Due to the anatomical complexity of the thoracic wall, the multiple attachments of the muscles to the ribs create a complex set of forces exerted on each rib during both respiration and movement. The directions of forces are distributed along the ribs and result in different lines of strain across the ribs. Rib fractures can alter both immediate respiratory function, muscular function, and long-term healing. Optimization of the local microenvironment is key in fracture healing, of which a large portion involves fracture stabilization. SSRFs gives the fractured bone segments a compressed, rigid environment to promote direct bone healing. A factor that influences proper healing is strain, the amount of movement at the fracture site. Too much or too little strain can contribute to improper bone healing and abnormal callus formation [6]. In our study, most non-union cases occurred between ribs 8 and 10. We suspect this is secondary to the multitude of varying forces on these areas listed above that do not get appropriately transmitted when there is a fracture along the site. Moreover, recent cadaveric studies suggest ribs 9 and 10 are often not attached anteriorly, making the segments more susceptible to flail physiology when injured [8,9]. Together, the unbalanced forces and peculiar anatomy may produce an environment that is suboptimal for healing. These clinical findings, in conjunction with recent anatomic studies, should be further evaluated with clinical and basic science evaluations.

Heterotopic ossification is the formation of bone in an abnormal soft-tissue location with trauma or surgery, often noted as the initiating event [10]. Although it is increasingly documented in the orthopedic literature, little is known about its pathogenesis [11]. Patients may present with localized pain, tenderness, and swelling around the affected area. Additionally, HO can cause limitations in range of motion as well as peripheral nerve entrapment, leading to disability in patients that develop HO [12]. This decreased range of motion, as well as nerve entrapment, may lead to a restrictive respiratory effort manifest as a limited ability to take a deep breath or increased respiratory rate. HO may not be readily apparent on radiographs, making the pathology even more difficult to research [10]. Little is known about the incidence and factors contributing to HO formation after rib fractures. Forty-three percent of patients in this study were found to have evidence of HO on follow-up CT imaging, and many of these were not evident on chest X-ray. The relationship between HO, timing of surgery, types of screw fixation, and year of operation was significant. However, these variables are highly related due to changes in practice patterns over time. During the early time of this evaluation, SD screws were not available. Additionally, surgery was often delayed until proof of failure to wean from the ventilator was established. Also, many of these patients had severely displaced fractures that likely had more soft tissue injury to the chest wall. The evolution of indications for SSRFs as well as maturation of the chest injury program over time may have influenced the incidence of HO formation. It is unclear which of these factors contribute to the formation of HO. Increased tissue trauma from more displacement of the rib fractures, as well as prolonged critical illness, may contribute to HO formation. Additionally, the authors hypothesize that the method of screw fixation may also be the source of HO, with nearly 90% of those affected undergoing fixation with self-tapping screws. The placement of self-tapping screws required bicortical drilling with a surgical drill. This drilling may distribute bone marrow and cortical bone fragments in the surgical field, with potential embedding into nearby soft tissue. These microscopic bone fragments may implant into the soft tissue and begin to form new bone in non-anatomic locations. Additionally, medications like non-steroidal anti-inflammatory drugs and bisphosphonates may be used to prevent HO formation [13]. All patients in this study were treated with non-steroidal anti-inflammatory drugs during their initial trauma. The authors are pursuing a multi-center study looking at the incidence of HO with different plating technologies that do and do not use drilling as part of the surgical repair.

Limitations of our investigation involve the small patient population and factors related to the rib plating process, particularly the type of plates used in these patients. All plates utilized in this study were from a single manufacturer. This study utilized a convenience sample of patients that were imaged for unrelated reasons. The small sample size limits the statistical power of this study. Additionally, being from a single center may introduce unmeasured or identifiable influences on outcomes that could bias the ultimate results. These findings should be evaluated in a multi-center evaluation using different rib fixation implants to account for the bias present in this evaluation. The clinical significance of the HO and non-union rib fractures could not be determined or measured. It is unknown if these patients have any symptoms from these findings and should be investigated in future evaluations of HO formation. In terms of HO, identification was likely underestimated due to the timeline in which it develops, with the recent literature suggesting the process may take up to years to develop [10,11]. This may also alter the association of HO and surgeries that were performed longer ago as there is a potential time-related follow-up bias. The diagnosis of HO was determined by the study team, although inter- and intra-observer reliability were not assessed. Lastly, we offer a theory that HO may be related to the technique used in SSRFs. This should be tested in animal models, as well as the histological evaluation of HO excisions, to determine if this theory is correct.

## 5. Conclusions

This investigation begins to elucidate the healing process of rib fractures following surgical fixation, offering valuable insights into the anatomical and pathological nuances of recovery. Notably, the data demonstrate that non-union is an uncommon complication, with occurrences primarily concentrated in the posterior and paraspinal segments of ribs 8 through 10. This anatomical distribution suggests that certain rib locations may be more prone to impaired healing, potentially due to biomechanical stressors, limited vascular supply, or difficulty in achieving optimal surgical stabilization in these regions.

In addition, our findings highlight a relatively high incidence of heterotopic ossification (HO), which was detected in over 40% of patients during follow-up CT imaging. The presence of HO raises concerns not only for its frequency but also for its potential to contribute to persistent pain, reduced thoracic mobility, and long-term functional limitations. Despite being a well-documented phenomenon in other orthopedic procedures, the mechanisms driving HO formation in rib fixation remain poorly understood and warrant further scrutiny.

Given the discomfort and morbidity associated with both non-union and HO, these results emphasize the clinical importance of identifying modifiable risk factors and preventive strategies. Future investigations should aim to delineate the impact of surgical timing, fixation techniques, patient-specific characteristics, and perioperative management on these complications. A well-designed multi-center study would provide the statistical power and diversity necessary to assess these variables comprehensively, ultimately guiding evidence-based practices that enhance patient outcomes and minimize long-term complications following rib fracture fixation.

## Figures and Tables

**Table 1:** Heatmap of the rib number and location of injuries. The incidence of fractures is color-coded, with lowest values in green and highest values displaying increasing red hues. The rows separate the ribs by number, 1–12. The columns are separated by anatomic region (CC—costal cartilage; A—anterior; L—lateral; P—posterior; PS—paraspinal).

Location of Fractures					
	CC	A	L	P	PS
1	1	0	1	1	1
2	1	0	4	2	1
3	1	3	5	4	1
4	1	4	5	4	2
5	1	5	9	7	4
6	1	4	10	10	4
7	0	4	11	11	5
8	0	3	10	11	6
9	0	1	8	10	7
10	0	1	4	6	6
11	0	0	2	2	1
12	0	0	0	2	0
**Total**	6	25	69	70	38

**Table 2:** Heatmap of the rib number and location of non-union fractures. The incidence of fractures is color-coded, with lowest values in green and highest values displaying increasing red hues. The rows separate the ribs by number, 1–12. The columns are separated by anatomic region (CC—costal cartilage; A—anterior; L—lateral; P—posterior; PS—paraspinal).

Non-Union of Fractures					
	CC	A	L	P	PS
1	0	0	0	0	0
2	0	0	1	0	0
3	0	0	0	0	0
4	0	0	0	0	0
5	0	0	0	0	0
6	0	0	2	0	0
7	0	0	0	0	0
8	0	0	0	1	0
9	0	0	0	3	0
10	0	0	0	1	2
11	0	0	0	0	0
12	0	0	0	0	0
Total	0	0	3	5	2

## Data Availability

The data presented in this study are available on request from the corresponding author due to institutional privacy restrictions.
